# Spatial and temporal distribution of reported dengue cases and hot spot identification in Quezon City, Philippines, 2010–2017

**DOI:** 10.1186/s41182-023-00523-x

**Published:** 2023-05-25

**Authors:** John Robert C. Medina, Rie Takeuchi, Chris Erwin G. Mercado, Calvin S. de los Reyes, Rolando V. Cruz, Melvin D. R. Abrigo, Paul Michael R. Hernandez, Fernando B. Garcia, Mika Salanguit, Ernesto R. Gregorio, Shin’ya Kawamura, Khew Ee Hung, Masami Kaneko, Daisuke Nonaka, Richard J. Maude, Jun Kobayashi

**Affiliations:** 1 Institute of Health Policy and Development Studies, National Institutes of Health, University of the Philippines Manila, 623 Pedro Gil St, Ermita, Manila, 1000 Metro Manila Philippines; 2 Department of Health Policy and Administration, College of Public Health, University of the Philippines Manila, 625 Pedro Gil St, Ermita, Manila, 1000 Metro Manila Philippines; 3 Department of Global Health, Graduate School of Health Sciences, University of the Ryukyus, 207 Uehara, Nishihara-Cho, Nakagami-Gun, Okinawa, 903-0215 Japan; 4 Graduate School of Public Health, International University of Health and Welfare, 4-3, Kodunomori, Narita, Chiba 286-8686 Japan; 5 Mahidol-Oxford Tropical Medicine Research Unit, Faculty of Tropical Medicine, Mahidol University, 420/6 Rajvithi Road, Bangkok, 10400 Thailand; 6 College of Arts and Sciences, University of the Philippines Manila, Padre Faura St., Ermita, Manila, 1000 Metro Manila Philippines; 7 Quezon City Epidemiology and Surveillance Unit, Local Government of Quezon City, Quezon City, Philippines; 8 Department of Environmental and Occupational Health, College of Public Health, University of the Philippines Manila, 625 Pedro Gil St, Ermita, Manila, 1000 Metro Manila Philippines; 9 Department of Health Promotion and Education, College of Public Health, University of the Philippines Manila, 625 Pedro Gil St, Ermita, 1000 Manila, Metro Manila Philippines; 10 Chubu Institute for Advanced Studies, 1200 Matsumoto-Cho, Kasugai, Aichi 487-8501 Japan; 11 Department of Biosphere and Environmental Sciences, Rakuno Gakuen University, 582 Bunkyodaimidoricho, Ebetsu-Shi, Hokkaido, 069-8501 Japan; 12 Centre for Tropical Medicine and Global Health, Nuffield Dept of Medicine, University of Oxford, Oxford, OX3 7FZ UK

**Keywords:** Dengue, Philippines, GIS, Hot spot, Spatial cluster, Quezon City, Getis-Ord General G statistic, And Getis-Ord Local Gi*

## Abstract

**Background:**

Dengue remains a major public health problem in the Philippines, particularly in urban areas of the National Capital Region. Thematic mapping using geographic information systems complemented by spatial analysis such as cluster analysis and hot spot detection can provide useful information to guide preventive measures and control strategies against dengue. Hence, this study was aimed to describe the spatiotemporal distribution of dengue incidence and identify dengue hot spots by *barangay* using reported cases from Quezon City, the Philippines from 2010 to 2017.

**Methods:**

Reported dengue case data at *barangay* level from January 1, 2010 to December 31, 2017 were obtained from the Quezon City Epidemiology and Surveillance Unit. The annual incidence rate of dengue from 2010 to 2017, expressed as the total number of dengue cases per 10,000 population in each year, was calculated for each *barangay*. Thematic mapping, global cluster analysis, and hot spot analysis were performed using ArcGIS 10.3.1.

**Results:**

The number of reported dengue cases and their spatial distribution varied highly between years. Local clusters were evident during the study period. Eighteen *barangays* were identified as hot spots.

**Conclusions:**

Considering the spatial heterogeneity and instability of hot spots in Quezon City across years, efforts towards the containment of dengue can be made more targeted, and efficient with the application of hot spot analysis in routine surveillance. This may be useful not only for the control of dengue but also for other diseases, and for public health planning, monitoring, and evaluation.

**Supplementary Information:**

The online version contains supplementary material available at 10.1186/s41182-023-00523-x.

## Introduction

With an active transmission in at least 128 countries and four billion people at risk, dengue is recognized as the most common mosquito-borne viral disease [1]. There have been at least 50 million apparent cases every year; the majority of which were reported in the Western Pacific region [2–4]. About 10,000 deaths per annum have been attributed to symptomatic dengue infection; most which were reported from the Southeast Asian region [3]. Since the epidemic of hemorrhagic fever in 1954, dengue remains a major public health concern in the Philippines [5]. The country was identified as one of the member states of the Association of Southeast Asian Nations (ASEAN) with the highest burden of dengue [6]. In 2022, more than two hundred thousand dengue cases were documented in the country, which was more than double the documented cumulative number of cases in the past 2 years [7]. Central Luzon (Region II), the National Capital Region (NCR), and the Cagayan Valley (Region II) were the regions with the highest magnitudes of reported cases in 2022 [8].

The Philippines is implementing its National Dengue Prevention and Control Program (NDPCP), aiming to reduce dengue morbidity through its six component strategies—surveillance, case management and diagnosis, integrated vector management, outbreak response, health promotion and advocacy, and research [9]. By offering timely detection of outbreaks and better collection of data on disease burden, a strong case surveillance system can offer supplementary yet vital contributions to achieve the aim of the NDPCP.

The utility of geographic information system (GIS) technology and spatial analytical techniques in dengue epidemiology and surveillance have already been demonstrated in several studies [10]. Global clustering analysis and local cluster detection of diseases are common spatial statistical methods that assess if the geographically bounded aggregation of a disease is of sufficient size and concentration that it is unlikely to be attributable to chance [11, 12]. In a study done in two cities and one county in Taiwan, global clustering analysis and local cluster detection showed that the dengue outbreaks in 2014 and 2015 were highly aggregated and the dengue hot spots were located in the urban, metropolitan districts [13]. Hot spot analysis of dengue in Singapore from 2013 to 2015 showed that the incidence was higher in blocks of low-rise houses, which paved the way for the recommendation of incorporating control of vector borne diseases in public housing plans [14]. The utility of GIS and spatial analysis in surveillance were further underscored during the pandemic when several web-based dashboards were developed to map the spatial distribution of COVID-19 cases globally and across subnational areas [15]. The COVID-19 pandemic in 2020 made it difficult for public health decision makers and managers to allocate human and financial resources and to render health care services against diseases with competing interests, e.g., COVID-19 and dengue. Hot spot identification became useful for identifying priority areas that need immediate attention.

There have been only few studies on mapping and detecting dengue hot spots in the Philippines. In the study of Garcia and de las Llagas in 2011, dengue incidence from 2005 to 2008 across the villages of Quezon City (QC) were mapped using ArcGIS and the maps were analyzed by overlaying with maps of population density, river networks, and land use [16]. The findings of the study provided useful insights into the relationship of dengue incidence with these factors; however, the conclusions were based on visual inspection that may be subjective. A later study, which covered the entire NCR including QC, mapped dengue incidence from 2010 to 2013 across the cities and municipality, and also compared the performance of local Moran’s I and Kulldorf’s spatial scan statistics for detecting dengue hot spots [17]. However, the analysis could have been achieved at a finer spatial scale, i.e., at the level of villages (called *barangay*).

In consideration of the abovementioned studies on dengue and the persistence of dengue as a public health problem in QC, a collaboration was established between the authors and the Quezon City Epidemiology and Surveillance Unit (QCESU) to demonstrate how GIS and hot spot detection can be used to study the local spatial epidemiology of the disease [18]. Specifically, the spatiotemporal distribution of dengue incidence from 2010 to 2017 was described and dengue hot spots were identified at the *barangay* level. Findings of this study could be useful for informing public health decision making in QC.

## Methods

### Study area

**Fig. 1:**
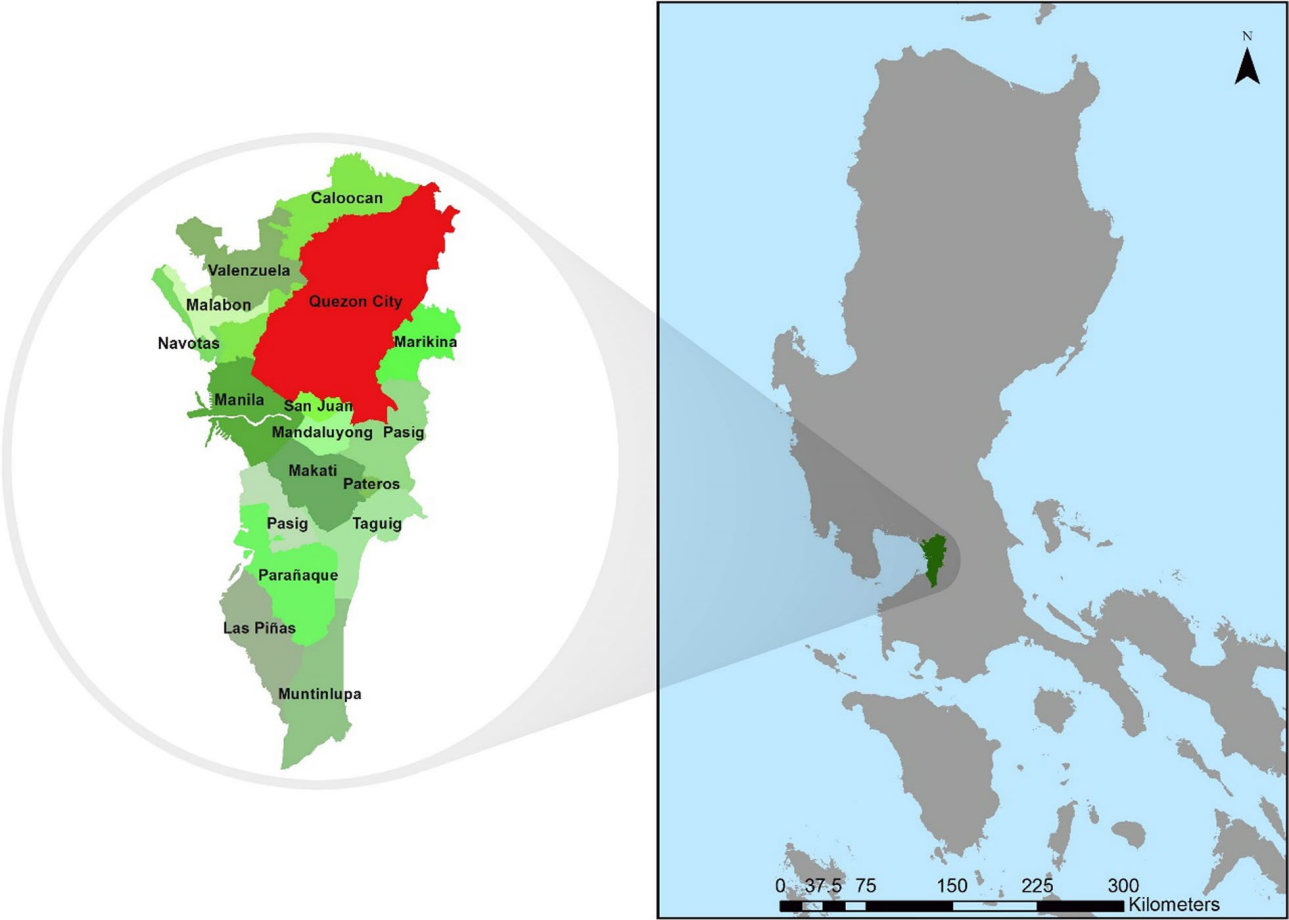
Reference map of Quezon City in the National Capital Region, Philippines. Quezon City is one of the 16 highly urbanized cities in the National Capital Region

**Fig. 2:**
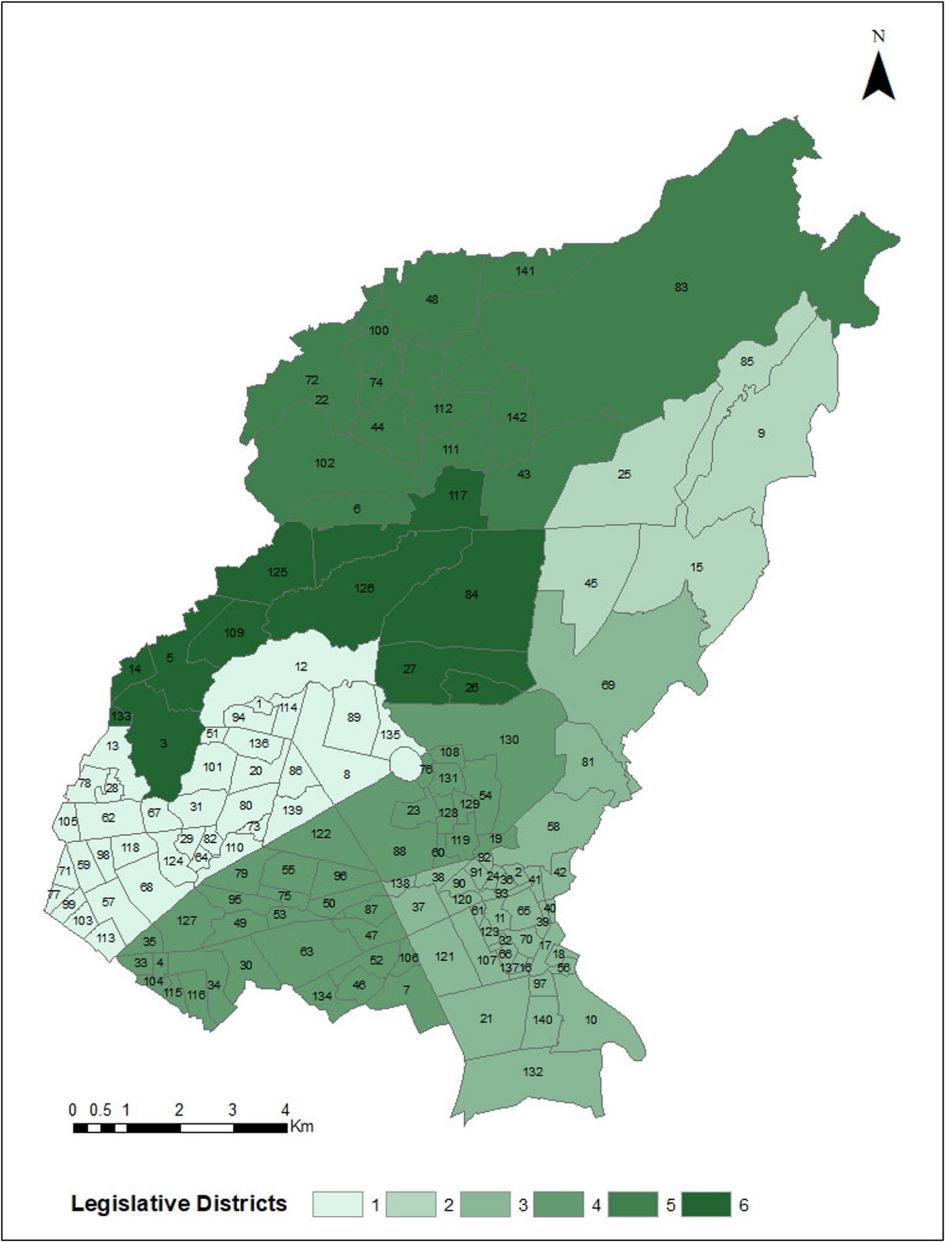
Six legislative districts and *barangays* of Quezon City, National Capital Region, Philippines. Quezon City is subdivided into 142 *barangays*, which are grouped into six legislative districts (Additional file 1)

Covering almost one-fourth of the northern part of the NCR, QC rests on the Guadalupe plateau (Fig. 1). Lowlands and rolling ridges characterize the city terrain. The elevation increases towards the north. QC has a total land area of 171.1 km^2^, the majority of which are residential areas. Since it is a highly urbanized area, much of the land area is used for industrial, commercial, institutional, and recreational purposes. In terms of political administration, QC is divided into six legislative districts and 142 *barangays* (Fig. 2). The *barangay* is the smallest administrative unit in the Philippines. The city has a population of 2,936,116, growing at a rate of 1.17% per annum. The population density in the city is 17,099 persons per km^2^ [19]. The city government is implementing a local dengue control program, which is complemented by a GIS-enabled epidemiology and surveillance unit.

### Data collection

Individual level deidentified data on reported dengue cases from January 1, 2010 to December 31, 2017 was provided by the QCESU. The data included the date of consultation, *barangay* of residence, and name of the disease reporting unit (DRU). Under the Philippine Integrated Disease Surveillance and Response, all units of health service delivery rendered as DRUs in the city, i.e., *barangay* health stations, hospitals, and clinics, are mandated to routinely submit weekly reports of all dengue cases to QCESU [20]. The population data from each *barangay* for the national census years 2010 and 2015 were obtained directly from the Civil Registry Department of the Local Government of Quezon City. Population estimation and projection were done for the years 2011 to 2014 and 2016 to 2017 based on the data from 2010 and 2015, respectively.

### Data analysis

The annual incidence rate of dengue from 2010 to 2017, expressed as the total number of dengue cases per 10,000 population in each year, was calculated for each *barangay*. Thematic mapping, global cluster analysis, and hot spot analysis were performed using ArcGIS 10.3.1 (Environmental Systems Research institute, Inc., Redlands, CA, USA). Global cluster analysis was performed using the Getis-Ord General G statistic, while hot spot analysis was done using Getis-Ord Local Gi* statistic. Both tests were performed at the 95% confidence level. Global clustering and identification of dengue hot spots were both indicated by a statistically significant, positive Gi* index [21].

### Ethical considerations

Since this study utilized secondary data that did not include any personally identifiable information, an exemption from review and subsequent approval for implementation was granted by the Far Eastern University—Nicanor Reyes Medical Foundation Institutional Ethics Review Committee (FEU-IERC Code: 2018-0011) and the Ethical Committee of University of the Ryukyus (Approval Number: 1391).

## Results

### Dengue cases and incidence rate

From 2010 to 2017, a total of 41,045 reported dengue cases were recorded by the QCESU. The 8-year average of the annual dengue incidence rates in QC was 16.7 per 10,000. There was large variation in the magnitude of reported cases across the years. The highest number of cases and incidence rate were reported in 2012, while the lowest were in 2011 (Table 1).

**Table 1:** Annual dengue cases and incidence in Quezon City, Philippines, 2010–2017

	2010	2011	2012	2013	2014	2015	2016	2017
Number of cases	2974	1815	9714	6203	2420	6473	4459	7527
Incidence (per 10,000)	10.8	6.5	32.5	21.7	8.4	22.0	15.0	24.9

### Spatial distribution

**Fig. 3:**
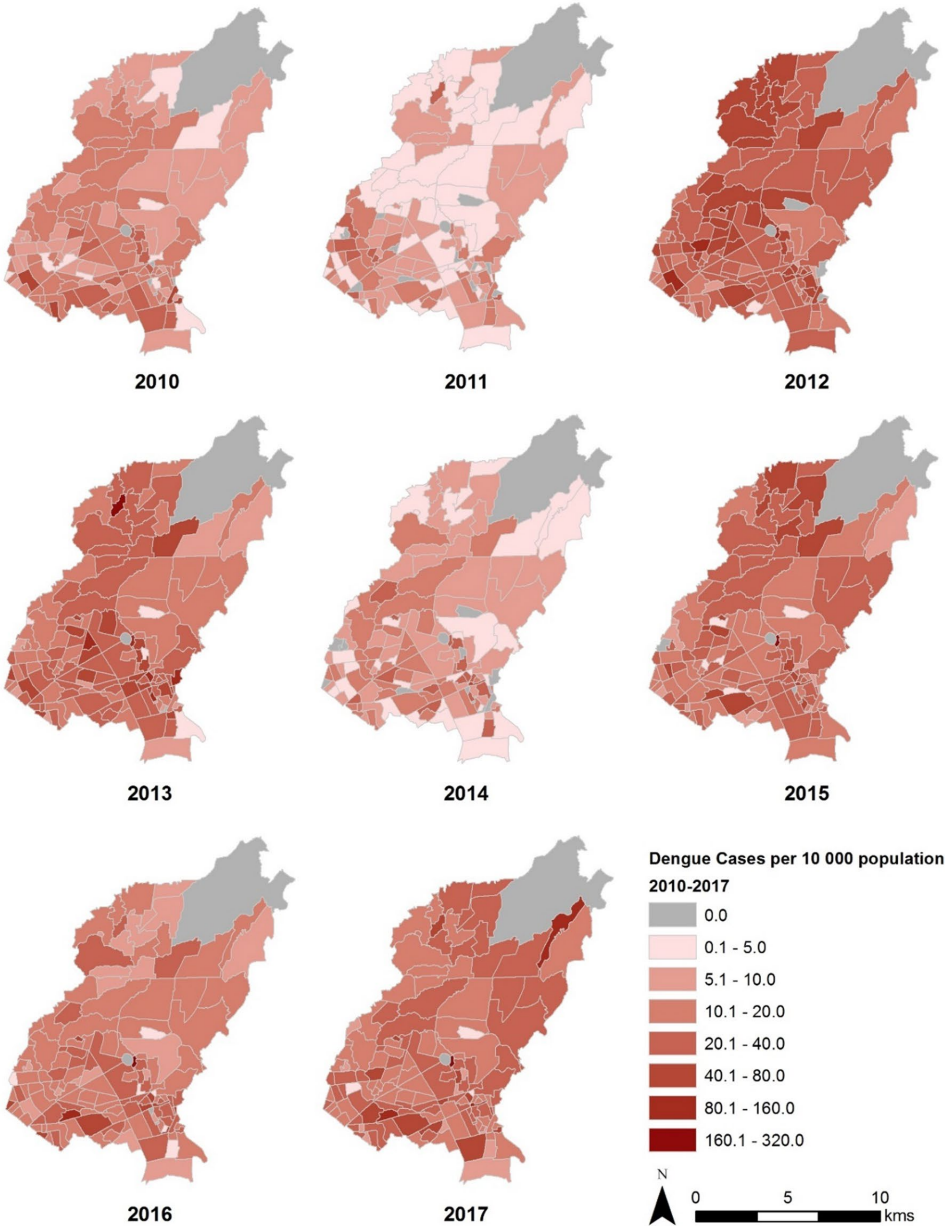
Numbers of reported dengue cases per 10,000 population in Quezon City, Philippines, 2010–2017

The spatial distribution of reported dengue cases in QC varied highly across years, as presented in incidence rate maps (Fig. 3). In 2010, dengue was present in almost the entire study area. Only four *barangays* (*barangay* 32, 40, 61, and 92) had no reported cases of dengue. The highest incidence rate at 67.44 cases per 10,000 people was documented in *barangay* 17.

In 2011, dengue appeared to concentrate in the South and near the boundaries of the central area of QC. In the same year, almost 10% of all the *barangays* had no reported cases of dengue. The highest incidence rate at 39.93 cases per 10,000 people was documented in *barangay* 62.

The highest number of dengue cases from 2010 to 2017 was reported in 2012 with more than 9000 cases. Dengue was reported in almost all of the *barangays* in that year. Only *barangay* 18, 26, and 42 had no reported cases of dengue. The highest incidence rate at 106.84 per 10,000 was documented in *barangay* 76.

Although the total number of reported dengue cases decreased in 2013, some *barangays* had higher incidence when compared to others. *Barangay* 74 had the highest incidence at 185.79 per 10,000. Only *barangay* 16 had no reported dengue cases.

In 2014, 20% of *barangays* had an incidence rate of reported dengue cases less than 5 per 10,000. About 8.5% of all the *barangays* had no reported dengue cases. The highest incidence rate was reported from *barangay* 93 with 60.94 per 10,000.

An increase in the magnitude of disease was observed in 2015. Only *Barangay* 61 and 78 had no reported dengue cases. *Barangay* 76 had the highest incidence rate at 176.17 per 10,000.

In 2016 and 2017, the spatial distributions of reported dengue cases were almost comparable. In both years, barangays 48 and 76 had the highest incidence rate. *Barangays* 32 and 61 had no reported dengue cases in 2016. However, in 2017, all *barangays* had reported dengue cases.

### Cluster analysis

**Fig. 4:**
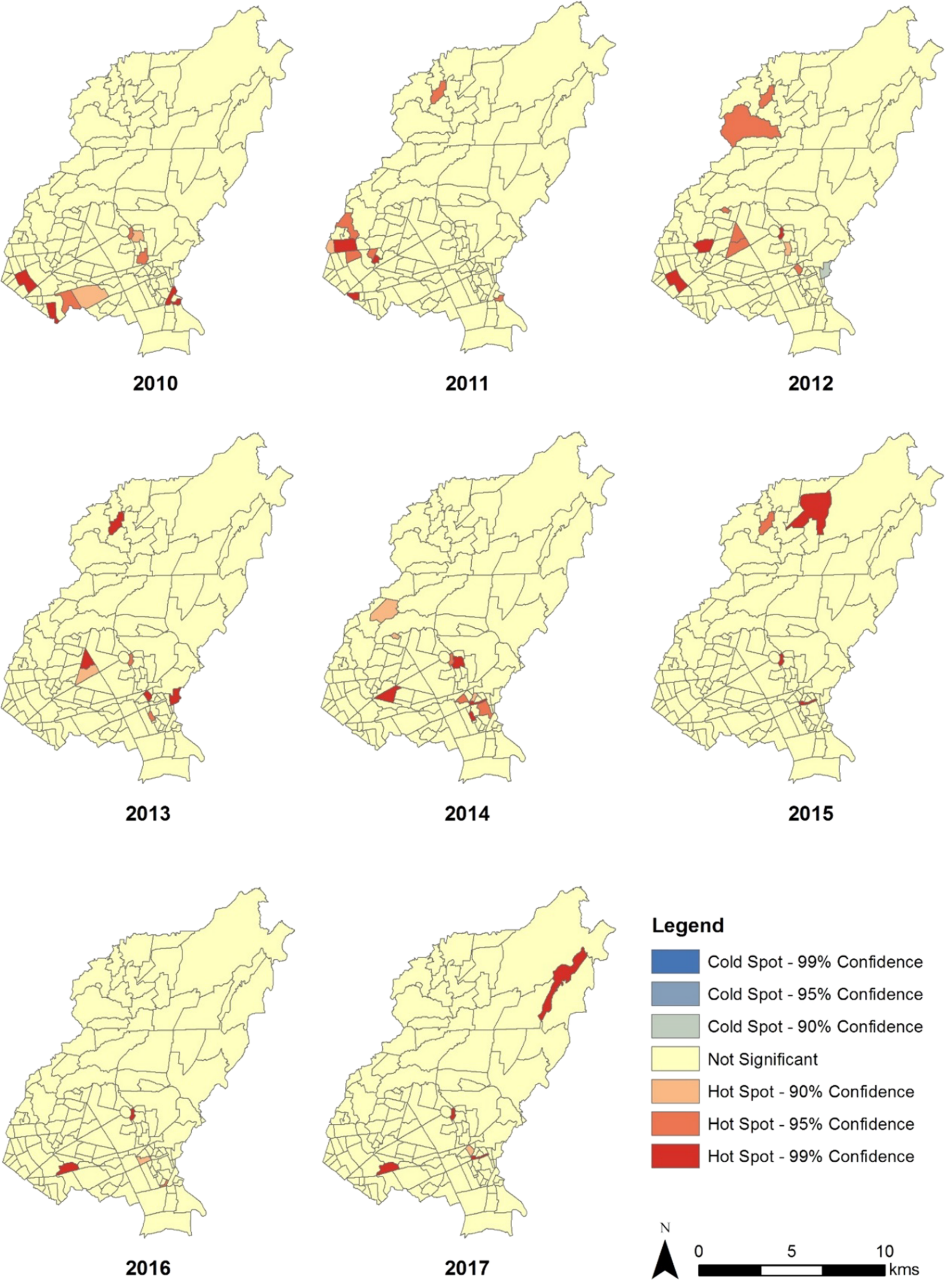
Dengue incidence rate hot spots identified through the Getis-Ord local Gi* statistic

Statistically significant global clustering of reported dengue cases was observed from 2011 to 2015 (Table 2). Local clusters were evident from 2010 to 2017 (Fig. 4). The highest count of identified hot spots was in 2012 with nine hot spots, while the lowest was in 2016 with only three hot spots. From 2010 to 2017, 18 *barangays* were identified as hot spots. *Barangay* 76 was identified annually as a hot spot, except in 2011. *Barangay* 74 was identified as a hot spot consecutively from 2011 to 2013 and then in 2015. *Barangay* 93 was identified as a dengue hot spot in the years 2014, 2015 and 2017. Other *barangays* that were identified in consecutive years were *Barangays* 56 (2010 to 2011), 86 (2012 to 2013), 91 (2012 to 2013), 123 (2014 to 2015), and 49 (2016 to 2017).

**Table 2:** Results of global clustering analysis, 2010–2017

Years	General G		*z*_G_-score	*p* value
	Observed	Expected		
2010	0.0071	0.0070	1.3507	0.1768
2011	0.0078	0.0070	4.6257	< 0.0001
2012	0.0075	0.0070	4.6769	< 0.0001
2013	0.0074	0.0069	3.0645	0.0022
2014	0.0076	0.0070	2.7683	0.0056
2015	0.0112	0.0070	7.2356	< 0.0001
2016	0.0071	0.0070	0.9625	0.3358
2017	0.0070	0.0070	− 0.1094	0.9129

## Discussion

In the current study, the annual spatial and temporal variations in the distribution of reported dengue cases in QC from 2010 to 2017 were investigated through mapping, cluster analysis, and hot spot identification. The results revealed that the incidence rates of dengue across *barangays* were spatially heterogeneous and the dengue hot spots were unstable and varied over the years.

Significant clustering of dengue incidence was observed in QC from 2011 to 2015 based on the results of the global clustering analysis using the Getis-Ord General G statistic. The observed patterns in 2010, 2016, and 2017 were attributable to random processes. However, local cluster detection using Getis and Ord Local Gi* statistic identified hot spots or clusters of areas with high incidence rates throughout the study period. The discrepancy can be explained by the limitation of global measures of spatial autocorrelation, such as the General G statistic. Those measures observe the assumption that spatial processes are stationary, thereby local spatial processes are obscured [11].

To the best of the authors’ knowledge, this is the first study in QC that mapped the incidence of dengue and detected hot spots over time. The presence of time varying hot spots is evidence of spatial heterogeneity, which indicates the non-uniform distribution of any attribute value in a geographic area, i.e., dengue incidence across *barangays* of QC [11, 22]. Across the years, different hot spots were identified except for a minority of *barangays* that remained consistently as hot spots, e.g., *barangay* 76. Mapping the stability of hot spots proved to be useful, because stable hot spots can predict the occurrence of future hot spots and can identify areas that are highly susceptible for a targeted implementation of interventions [23]. In the current study, most of the identified hot spots were unstable. Having hot spot instability has implications for the public health response towards controlling and preventing dengue. Since new hot spots were identified across the years, data collection and analysis need to be done quickly each year to guide targeting of control activities. Ideally, near real-time data collection and analysis are required to rapidly identify new hotspots in a particular year as part of an early warning system to give the public health authorities enough time to respond. This could be operationalized on a weekly or monthly basis. The response should be designed dynamically and contextualized to the situation of the identified hot spot. Aside from depending on the proactive approach of dengue control and prevention, the reactive arm should be strengthened.

Another implication of observing hot spot instability is the identification of the causes of the variability across years. The current study was not designed to determine the factors driving the spatial heterogeneity and instability of hot spots. However, cautious observations can be made. Throughout the study period, the dengue hot spots were frequently situated in the southern half of the city, particularly those barangays in Districts 3 and 4. Having an elevation of 50 m at most, these areas are the city’s lowlands. The predilection of *Aedes aegypti* to thrive in urban lowlands can explain the observed clustering of dengue in the south [24]. Specifically, the consistent clusters were situated in residential areas that are near commercial areas, which was also noted in a previous study done by Garcia and de las Llagas [16]. Other factors that had been suggested by their study that may explain the distribution of dengue incidence in QC were the presence of informal settlements, poor sanitation, and soil organic carbon content in the area [16]. However, the association of these factors with the observed spatial pattern of dengue in this study was not examined. Spatial regression could be done, provided suitable covariate data could be obtained; i.e., data with the same spatial and temporal scales [22].

During the first 2 years of the COVID-19 pandemic, an apparent decline in reported dengue cases was observed in several countries including the Philippines [25, 26]. This could be the consequence of implementing public health and social measures that restricted human mobility, such as community quarantine, school closures, and lockdowns [27]. However, essential health services were substantially disrupted as countries focused on strengthening their COVID-19 responses [28, 29]. Diagnostic and surveillance capacities of countries could have been limited by the pandemic, which may have resulted in the underreporting of other diseases including dengue [26]. The decline in reported dengue cases may have also been due to reporting hesitancy due to people’s fear of acquiring COVID-19 upon visiting a health facility [30]. Anticipated stigma associated with COVID-19 could have also led people to avoid being tested, which could hamper case detection of both COVID-19 and dengue [31]. As the Philippines moves towards the relaxation of countermeasures against COVID-19, outbreaks of dengue may occur [32, 33]. Spikes in the number of reported dengue cases had already been documented in certain regions of the Philippines, such as the Zamboanga Peninsula, Cagayan Valley, Western Visayas, and Davao Region [34].

While COVID-19 continues to be a priority, resources may remain unevenly allocated for the control and prevention of other diseases including dengue. To address this, hot spot analysis can be used to identify high priority areas and inform public health decision making. However, the instability of hot spots indicates that the analysis needs to be updated more often. This opens an opportunity for integrating hot spot analysis as an innovation in routine dengue surveillance, which can be institutionalized in every municipality or city in the country though a national policy.

## Conclusions

The current study demonstrated the utility of GIS, global cluster analysis and hot spot identification in assessing the dengue situation in QC. Findings revealed that dengue remains a challenge to the health situation in QC considering the spatial heterogeneity and the instability of dengue hot spots. Through the identification of hot spots, efforts towards the containment of dengue can be made more targeted and efficient.

## Supplementary Information

**Additional file 1. **Barangays in Quezon City, the Philippines.

## Data Availability

The data that support the findings of this study are available from the QCESU, but restrictions apply to the availability of these data, which were used under permission for the current study, and so are not publicly available. Data are, however, available from the authors upon reasonable request and with permission of the QCESU.

## Abbreviations

ASEAN: Association of Southeast Asian Nations
COVID-19: Coronavirus disease 2019
DOH: The Department of Health, the Philippines
DRU: Disease Reporting Unit
GIS: Geographic information system
NDPCP: National Dengue Prevention and Control Program
QC: Quezon City
QCESU: The Quezon City Epidemiology and Surveillance Unit
WHO: World Health Organization
