# Supplementary material for: Spatial and temporal distribution of reported dengue cases and hot spot identification in Quezon City, Philippines, 2010–2017
Source: Trop Med Health. 2023 May 25;51:31. doi: 10.1186/s41182-023-00523-x (PMC10208904; doi:10.1186/s41182-023-00523-x)
Supplement: Supplementary file 1 — Additional file 1. Barangays in Quezon City, the Philippines. [file 41182_2023_523_MOESM1_ESM.docx]

| **Barangay Code** | **Name of Barangay** |
| --- | --- |
| 0 | Alicia |
| 1 | Amihan |
| 2 | Apolonio Samson |
| 3 | Doña Aurora |
| 4 | Baesa |
| 5 | Bagbag |
| 6 | Bagong Lipunan ng Crame |
| 7 | Bagong Pag-Asa |
| 8 | Bagong Silangan |
| 9 | Bagumbayan |
| 10 | Bagumbuhay |
| 11 | Bahay Toro |
| 12 | Balingasa |
| 13 | Balong Bato |
| 14 | Bayanihan |
| 15 | Blue Ridge A |
| 16 | Blue Ridge B |
| 17 | Botocan |
| 18 | Bungad |
| 19 | Camp Aguinaldo |
| 20 | Capri |
| 21 | Central |
| 22 | Quirino 3B (Claro) |
| 23 | Culiat |
| 24 | Damar |
| 25 | Damayan |
| 26 | Damayang Lagi |
| 27 | Del Monte |
| 28 | Dioquino Zobel |
| 29 | Don Manuel |
| 30 | Doña Imelda |
| 31 | Doña Josefa |
| 32 | Duyan-Duyan |
| 33 | E. Rodriguez |
| 34 | East Kamias |
| 35 | Escopa 1 |
| 36 | Escopa 2 |
| 37 | Escopa 3 |
| 38 | Escopa 4 |
| 39 | Fairview |
| 40 | Gulod |
| 41 | Holy Spirit |
| 42 | Horseshoe |
| 43 | Immaculate Concepcion |
| 44 | Kaligayahan |
| 45 | Kalusugan |
| 46 | Kamuning |
| 47 | Katipunan |
| 48 | Kaunlaran |
| 49 | Kristong Hari |
| 50 | Krus Na Ligas |
| 51 | Laging Handa |
| 52 | Libis |
| 53 | Lourdes |
| 54 | Loyola Heights |
| 55 | Maharlika |
| 56 | Malaya |
| 57 | Mangga |
| 58 | Manresa |
| 59 | Mariana |
| 60 | Mariblo |
| 61 | Marilag |
| 62 | Masagana |
| 63 | Masambong |
| 64 | Santo Domingo |
| 65 | Matandang Balara |
| 66 | Milagrosa |
| 67 | N.S. Amoranto |
| 68 | Nagkaisang Nayon |
| 69 | Nayong Kaunlaran |
| 70 | Novaliches Proper |
| 71 | Obrero |
| 72 | Old Capitol Site |
| 73 | Paang Bundok |
| 74 | Pag-Ibig Sa Nayon |
| 75 | Paligsahan |
| 76 | Paltok |
| 77 | Pansol |
| 78 | Paraiso |
| 79 | Pasong Tamo |
| 80 | Payatas |
| 81 | Phil-Am |
| 82 | Pinagkaisahan |
| 83 | Pinyahan |
| 84 | Project 6 |
| 85 | Quirino 2A |
| 86 | Quirino 2B |
| 87 | Quirino 2C |
| 88 | Quirino 3A |
| 89 | Ramon Magsaysay |
| 90 | Roxas |
| 91 | Sacred Heart |
| 92 | Saint Ignatius |
| 93 | Saint Peter |
| 94 | Salvacion |
| 95 | San Agustin |
| 96 | San Antonio |
| 97 | San Bartolome |
| 98 | San Isidro Labrador |
| 99 | San Isidro |
| 100 | San Jose |
| 101 | San Martin De Porres |
| 102 | San Roque |
| 103 | San Vicente |
| 104 | Sangandaan |
| 105 | Santa Cruz |
| 106 | Santa Lucia |
| 107 | Santa Monica |
| 108 | Santa Teresita |
| 109 | Santo Cristo |
| 110 | Santo Niño |
| 111 | Santol |
| 112 | Sauyo |
| 113 | Sienna |
| 114 | Sikatuna Village |
| 115 | Silangan |
| 116 | Socorro |
| 117 | South Triangle |
| 118 | Tagumpay |
| 119 | Talayan |
| 120 | Talipapa |
| 121 | Tandang Sora |
| 122 | Tatalon |
| 123 | Teachers Village East |
| 124 | Teachers Village West |
| 125 | U.P. Campus |
| 126 | U.P. Village |
| 127 | Ugong Norte |
| 128 | Unang Sigaw |
| 129 | Valencia |
| 130 | Vasra |
| 131 | Veterans Village |
| 132 | Villa Maria Clara |
| 133 | West Kamias |
| 134 | West Triangle |
| 135 | White Plains |
| 136 | Pasong Putik |
| 137 | North Fairview |
| 138 | Greater Lagro |
| 139 | New Era |
| 140 | Batasan Hills |
| 141 | Commonwealth |
